# Reclaiming Land, Identity and Mental Wellness in Biigtigong Nishnaabeg Territory

**DOI:** 10.3390/ijerph19127285

**Published:** 2022-06-14

**Authors:** Elana Nightingale, Chantelle Richmond

**Affiliations:** Department of Geography and Environment, Western University, London, ON N6A 5C2, Canada; chantelle.richmond@uwo.ca

**Keywords:** indigenous wellness, indigenous mental health, environmental repossession, indigenous identity, Anishinaabe, connection to land, community-based research

## Abstract

Indigenous peoples globally are pursuing diverse strategies to foster mental, emotional, and spiritual wellness by reclaiming and restoring their relationships to land. For Anishinaabe communities, the land is the source of local knowledge systems that sustain identities and foster mino-bimaadiziwin, that is, living in a good and healthy way. In July 2019, the community of Biigtigong Nishnaabeg in Ontario, Canada hosted a week-long land camp to reclaim Mountain Lake and reconnect Elders, youth and band staff to the land, history, and relationships of this place. Framed theoretically by environmental repossession, we explore the perceptions of 15 participating community members and examine local and intergenerational meanings of the camp for mental wellness. The findings show that the Mountain Lake camp strengthened social relationships, supported the sharing and practice of Anishinaabe knowledge, and fostered community pride in ways that reinforced the community’s Anishinaabe identity. By exploring the links between land reclamation, identity, and community empowerment, we suggest environmental repossession as a useful concept for understanding how land reconnection and self-determination can support Indigenous mental wellness.

## 1. Introduction

Relationships to land are foundational to holistic and collective Indigenous approaches to mental wellness [1,2,3,4]. According to the Anishinaabe philosophy of mino-bimaadiziwin, living in a good and healthy way involves sustaining relationships of reciprocity and responsibility with all living things, including humans, animals, spirit, and future generations [5,6,7]. Through the individual and communal protocols required to maintain these relationships, the physical, mental, emotional, and spiritual wellness of communities is strengthened. In return, the land upholds its own responsibilities by providing the gifts necessary for living well. The land is thus more than a physical landscape or the location of material resources for survival [8]. It is the source of Indigenous knowledge that guides communities’ social, political, ceremonial, and everyday practices, and shapes Anishinaabe culture and identity [6,9].

The geographies of Indigenous health emerged out of the understanding that fostering wellness involves restoring and sustaining individual and community connections to land [10,11,12]. Drawing from population health and environmental health approaches, the growing discipline has documented the ongoing impacts of colonization and dispossession in which communities have been physically and politically displaced from their ancestral territories [13,14,15,16]. Legislation, policy, and development have aimed to limit the ability of families and communities to practice, teach, and learn Indigenous knowledge with implications for social support, identity formation, sense of belonging, and self-sufficiency [17,18,19]. More recently, the field has highlighted the strategies being developed by communities to foster self-determination over both land and wellness by drawing on their own strengths and skills [20,21,22]. The concept of environmental repossession seeks to describe these particular processes, and to explore how they are being implemented as a means to reclaim territory and reconnect with the knowledge, relationships, and identities tied to these places [23].

This paper presents a case study of environmental repossession in the territory of Biigtigong Nishnaabeg to explore and describe the impacts of these processes for community mental wellness. Building on a growing body of evidence around the role of repossession for supporting personal and collective relationships to land, Indigenous knowledge, and each other, we describe a community-led, land-based camp and the experiences of participating youth, adults, and Elders.

### 1.1. Environmental Repossession and Mental Wellness

The concept of environmental repossession emerged in response to a global movement of Indigenous communities seeking to reclaim their lands and ways of living for wellness [23]. Despite the impacts of colonization and persistent structures of oppression, Indigenous peoples are restoring their deep and multidimensional relationships to the land through diverse social, political, economic, and cultural processes [24,25]. Environmental repossession aims to describe these processes and to understand how they are being developed and implemented as a means to foster physical, mental, emotional, and spiritual wellness. As communities draw on their own histories, protocols, and knowledges to apply repossession, individual efforts will be place-based and adapted to each geographical and cultural context [22]. For instance, local processes of repossession can vary from canoe trips in the bush [26], to tree hugging in urban centres [21], to reoccupation protests [27].

While a wide body of literature has focused on the concept of Indigenous resilience in the face of colonial dispossession and trauma [28,29,30], environmental repossession shifts attention from strategies of Indigenous survivance and adaptation, to the revitalization of self-determination over land. By asserting Indigenous rights to land, communities strengthen their connections to the source of their knowledge systems, identities, and nationhood [31]. Access to land is essential for food security, physical activity, medicines, and environmental resources that support healing and physical wellbeing [12,16,32,33]. Equally important, however, the land provides the space for learning and teaching Indigenous knowledge through intergenerational relationships [3]. It is where families and communities gather to fulfill their responsibilities, practice their skills, and connect with each other in ways that foster confidence, belonging, pride, and social support [1,34]. Further, being on the lands of one’s ancestral territories simultaneously links community members with their ancestors and future generations, and reinforces the spiritual relatedness between humans and all of creation [4,35]. Reclaiming land is thus not only a physical process, but is multilayered with personal, family, and collective meanings that foster mental wellness.

The promise of environmental repossession lies in its flexibility and responsiveness to local needs and knowledge systems. Strategies of repossession are both developed and implemented by Indigenous communities themselves, based on their own experiences and understandings of mental wellness. As many Indigenous peoples in Canada continue to struggle with access to adequate and culturally appropriate mental health services [36,37,38], repossession holds potential as a community-based alternative. Alongside improving access to Indigenous-led counselling and interventions [39,40], these land-based processes may be used to support a holistic and long-term approach to Indigenous healing and wellness.

### 1.2. Land, Social Relationships and Indigenous Knowledge

Current evidence around the application of environmental repossession in community contexts suggests that access to land should be supported by strong social relationships and Indigenous knowledge to strengthen wellness. For example, Big-Canoe and Richmond’s [23] community-based study with Anishinaabe youth demonstrates that social relationships may be critical to restoring environmental connections for mental wellness. Their results emphasize the role of intergenerational relationships in sharing the Anishinaabe knowledge and skills that facilitate land use. The participating youth described how increasing land-based activities with Elders is necessary to both encourage healthy behaviours and reinforce Anishinaabe identity.

Similarly, Tobias and Richmond’s [22] participatory research with Anishinaabe Elders highlights intergenerational community gatherings and programs as important strategies of environmental repossession. While these activities may take different forms (e.g., youth camps, community garden, language workshops), the Elders describe a common focus on strengthening relationships with youth as a means to increase land use, improve health and healing, and foster community pride (p. 235). Experiences of dispossession have targeted not only land access, but also the family relationships through which the community’s land-based culture is shared and sustained. Local repossession efforts are therefore aimed at restoring social relationships and are intended to foster positive identity, social support, and sense of belonging among youth.

More recently, Mikraszewicz and Richmond [26] partnered with Biigtigong Nishnaabeg to examine how a community canoe trip functions as a strategy of repossession to reclaim significant sites in their ancestral territory. Interviews with trip participants suggest that the sharing of community history and Anishinaabe knowledge supported youth’s relationships to the land by creating a sense of place tied to both personal and family connections and cultural meanings. By being on the land together, space was created for the youth to strengthen their social relationships with Elders and each other, to gain confidence in their land knowledge and skills, and to build a personal relationship to their territory as a source of identity and wellness. 

### 1.3. Land Camps

Land-based or ‘bush’ camps are becoming a recognized model in Indigenous healing and mental wellness, with increasing scholarly attention given to the development, implementation, and evaluation of these programs [34,41,42]. While land camps differ greatly in content and structure across diverse geographies and cultures, they typically emphasize intergenerational connections and the sharing of Indigenous knowledge for social support, confidence, and belonging [1,43,44,45]. Through the teaching of land-based skills, such as hunting, food preparation and ceremony, camps intend to provide the time, space, and relationships for participants to reconnect with their identities and the land [3,46]. Drawing on Indigenous pedagogies, this teaching often takes place through sharing personal experiences, revitalizing language, and learning by doing to build individual self-esteem and independence [34,47]. By learning on and from the land, camps further aim to ground participants in their spiritual relatedness with the land as a teacher, relative, and source of support [48,49]. Generally, the intended participants are youth, particularly those who may be experiencing wellness challenges, and camp leaders are Elders or knowledge holders, although some camps may include Western-trained therapists for specific interventions. 

Community-led land camps seek to address the impacts of intergenerational trauma and displacement on relationships to the self, the family, the community, and the land [29,48,50]. In Canada, legislation and policy aimed to physically disconnect Indigenous peoples from their territories through the creation of reserve lands and forced relocation [51,52,53]. At the same time, the residential school system targeted the family relationships that sustain communities’ land-based knowledge systems and identities [54]. The shift to market-based economies and wage labour has furthered this disconnect by limiting the time available for family members to be on the land together [16,55]. As a result, many communities have expressed concerns about younger generations lacking the relationships, skills, and knowledge for land use, with consequences for both cultural continuity and wellness [22,49,56]. Land camps have evolved as a mechanism for individual and social healing by returning community members to the land and revitalizing the land-based languages, protocols, and responsibilities that uphold Indigenous identities. 

Given the potential for land camps to foster community healing, Radu, House, and Pashagumskum [3] argue that they can be understood as processes of local collective empowerment and decolonization. In line with Corntassel’s conceptualization of decolonization as an everyday and land-based practice [57], land camps allow Indigenous people to reaffirm their personal and collective values, social relations, responsibilities, and identities in the context of contemporary needs and challenges. This framing suggests that land camps may be important strategies of environmental repossession. As community members restore their relationships to land and each other, camps can become sites of political resistance and Indigenous resurgence through which local rights are asserted and processes of wellness are reclaimed [27,57]. 

Building on the scholarly literature around land connections and Indigenous mental wellness, this paper explores community meanings of a land camp to reclaim Mountain Lake in Biigtigong Nishnaabeg territory. Framed by the concept of environmental repossession, we document the perceptions of youth, adults, and Elders participating in the week-long camp and examine the implications for community relationships, knowledge, and pride. Specifically, the goal is to describe how strategies of repossession can support land reclamation, Anishinaabe identity, and wellness. 

## 2. Materials and Methods

The research presented here is framed by environmental repossession as both a theoretical concept and applied methodology within the geographies of Indigenous health and wellness [10]. In response to decades of pathologizing and extractive health research, Indigenous scholars and communities have advocated for increasing control over research frameworks and design [58]. Environmental repossession developed out of this methodological shift as it centers Indigenous knowledges, experiences, and strengths as a means to support self-determination over land and wellness [26]. Drawing from Indigenous research methods, repossession begins from the principle of relationality to emphasize Indigenous knowledge systems as the guiding methodological lens and to uphold community ownership over the entire research process, from determining objectives to dissemination [59,60,61]. This means that study design is geographically specific and develops out of the values, protocols, relationships, and goals of particular communities or groups. Most importantly, this means that applying repossession is a transformative approach in which research not only seeks to describe processes of land reclamation, but actively contributes to these efforts. 

This case in Biigtigong Nishnaabeg is part of an ongoing international study that brings together researchers and community partners in Canada, Aotearoa, and Hawai’i to explore the application of environmental repossession in local contexts. Starting from Indigenous methodologies allowed this research to explore the concept of environmental repossession through Biigtigong’s local ways of gathering, sharing, and validating knowledge, and within the context of the community’s social and environmental relationships. As an Anishinaabe community, Biigtigong’s local worldview and ways of knowing are specifically framed by Anishinaabe knowledge systems that encompass history, teachings, and spirituality, and emphasize the holistic philosophy of mino-bimaadiziwin, living a good life. In the context of research, Anishinaabe scholars Debassige [7], Bell [5], and McGregor [61] have argued that mino-bimaadiziwin can be applied as a methodological approach in which the researcher commits to both living in a good way and supporting Indigenous communities towards their own goals of a good and well life. In practice, this means that the foundation of research is the building of long-term relationships between the researcher and their community partner, as well as self, past and future, environment, Indigenous knowledge, and spirit [61]. This study is part of a long-standing research and personal relationship between the second author, an Anishinaabe scholar, and Biigtigong, their home community.

This study phase was a collaboration between the two academic authors and Biigtigong’s Department of Sustainable Development. In 2018, the community’s Band Manager and staff from the Department of Sustainable Development identified a need for research to explore Biigtigong’s connection to Mountain Lake and the western boundary of its territory, an area from which community members have been largely disconnected [20]. Through regular meetings with department staff, it was determined that the first author, a settler graduate student, and a community researcher, the Manager of Culture, Heritage and Tourism, would lead this research. The research design was approved by the Non-Medical Research Ethics Board of the researchers’ home institution and publicly launched in Biigtigong in October 2018. 

With the goal of increasing land use and awareness of history at Mountain Lake, the community researcher selected a land camp as an appropriate and participatory method that would bring community members to this site and facilitate the collection of knowledge from attending Elders and knowledge holders. The first author and community researcher worked closely to plan, organize, and deliver the land camp at Mountain Lake. To facilitate participation among Elders and youth, the camp was scheduled as a daily event for one week in July 2019, with attendees returning home each day. Elders and key knowledge holders were personally invited by the community researcher and transportation was provided. The Department’s summer students and staff were also directly invited, although the camp was open to all interested community members. As the camp intended to gather Anishinaabe knowledge and history, activities were planned to encourage intergenerational sharing and storytelling through a mix of both structured and flexible time. For instance, each day began with a workshop to learn cultural skills, such as moccasin and rattle making, and afternoons involved sharing stories around a campfire and other social activities, including swimming and canoeing on the lake [Table 1]. All meals were cooked and shared communally to teach food preparation and facilitate relationship building. Camp participation fluctuated daily between 30–50 community members and the final day took place indoors on Biigtigong’s reserve due to rain.

Throughout the camp, the stories and experiences of attendees were collected, including four story-based, conversational interviews with Elders and knowledge holders. To further examine community meanings of the camp as a process of environmental repossession, in-depth story-based interviews were conducted with the participating summer students (*n* = 6) and department staff (*n* = 5) the following week. All interviews were conducted by the first author based on a flexible interview guide developed with the community researcher around perceptions of the camp and community connections to land. The interviews were audio recorded with permission and complimented by notes and observations from the community researcher.

The recorded interviews were transcribed and analyzed thematically [62] through a three-stage process that involved: (1) inductive coding by the first author to generate preliminary themes; (2) collaborative interpretation with the community researcher and second author; and (3) deductive analysis by the first author across the three distinct age groups of participants (youth, staff, Elders) and in reference to the research objectives. Through steps 1 and 2, findings began to emerge around the role of the camp for community mental wellness, which were explored in detail in step 3. As the settler graduate student took the lead in data collection and analysis for this paper, it is important to acknowledge how their positionality and social, cultural, and geographical distance from Biigtigong limits both their relational and interpretative capacity. They will never be able to understand the intimate relationship between Biigtigong and its ancestral territory, nor its complex Anishinaabe knowledge system. Over the past five years of collaboration, the first author and community researcher have developed a close research and personal relationship that has facilitated ongoing discussion around roles, interpretation of findings, and appropriate representation of Anishinaabe experiences. Formal and informal member-checking with the second author and broader Department of Sustainable Development has also been ongoing to verify that local meanings are understood, and that key and sacred community knowledge is excluded from the research outputs. Given capacity and workload constraints on department staff, it was decided that the first author would be responsible for preparing dissemination outputs for both academic and community audiences, with continuous validation by the community researcher, second author and various department staff. 

## 3. Results

The results of our analysis are presented around three key findings in relation to individual experiences and community meanings of the Mountain Lake camp. As a strategy of environmental repossession, the land camp was a process of (1) strengthening social relationships; (2) sharing and practicing local Anishinaabe knowledge; and (3) restoring community pride. The findings below are discussed in relation to the three groups of participants at Mountain Lake (youth, staff, Elders) in order to understand generational perspectives around the role and impact of the camp for community members. 

### 3.1. Strenghthening Social Relationships

Although Biigtigong is a small community and many members are connected through family ties, participants expressed worry around the disconnection of intergenerational relationships. Elders, staff, and youth described how family and community relationships have been weakened by experiences of colonization, such as the residential and day school system and their related social impacts, as well as by contemporary work schedules and increasing technology use. As a result, youth explained that younger generations have less opportunities both to be out on the land and to connect with Elders and other knowledge keepers:

*Because a lot of people that I know, they’re in really broken homes and stuff like that. And that’s always been a struggle to those persons. They wanna go out hunting or canoeing, fishing and stuff like that, but they don’t have no one to go with. And give the kids opportunities that some kids don’t get to have. Because, usually, when we were out at Mountain Lake, I noticed that a lot of people that I work with, a lot of their relatives would be coming out. And I know my grandma came out one day, but since, I don’t know, that relationship isn’t there, it’s just like, oh she’s just another person in the crowd or whatever, that kind of thing. And it just hit that man, a lot of these families get to do this type of stuff together and I have to beg my family to do stuff with me.* (Youth 2)

At the start of the Mountain Lake camp, the participant groups indicated that they did not have close relationships with each other. In many cases, Elders and younger participants did not know each other, as one youth described:

*Some of [the Elders] didn’t even know who I was and they’re still talking to me. And then all of a sudden, they’re like, “Who’s your parents?” Then I’m like, “Oh, my god.” Then I’ll tell them and they’re like, “Oh, my gosh! I didn’t even recognize you.”* (Youth 3)

By bringing different generations together on the land, the camp provided the time and space for community members to begin rebuilding relationships in a relaxed and social environment. Through both structured and flexible activities, Elders, staff, and youth were able to socialize, share food and learn from each other.

*You know when I was at Mountain Lake, there was a few of them that I didn’t really talk to before and that I actually got to talk with. And like even [Knowledge Holder], like I’ve lived across from him for two years now and I barely even talk to him. I talked to him more that day than I did ever.* (Staff 5)

As this quote demonstrates, the camp created a special opportunity for community members to step outside of their daily routines and social expectations in order to spend time with the wider community. Another staff member highlighted how this opportunity to reconnect with community and family members on the land created an atmosphere of calm, relaxation, and emotional ease at the camp:

*When you come together in these kind of environments—people do have a good time. They have fun. It’s like whatever is going on in their lives for that moment, it’s gone. Because when you’re out there you have an opportunity, it’s almost like you’re cleansing your body when you’re on the land in an environment that you’re so familiar with. That’s what I noticed down there, people were just, they looked so at ease. They looked like they were relaxed. Was it being in the bush? Probably. Was it being around people that you don’t see very often? Probably, yes. But just taking the time and appreciating the environment, but also making those reconnections with family and friends and sharing tea and telling stories or doing activities that are going to make you forget life for a moment.* (Staff 4)

In particular, youth explained how activities, such as rattle making, which paired them up with Elders, provided a bridge across what can be an uncomfortable gap between the two generations:

*When you tell a youth to just go find an Elder and help an Elder, they get anxious. And then they get shy and they get intimidated, so they won’t go and help. So yeah, I think teaming them up with an Elder helps a lot. Because they haven’t been in that position before.* (Youth 1)

At the end of the camp, all participant groups reported that social connections were stronger, both personally and collectively. Elders described a new bond with the youth, that they had “made a new friend” and that each younger participant was “not just a youth, but now has a name.” The camp organizer summarized a hopeful sentiment: that these new relationships had created a sense of family and belonging among participants that would continue long after the week at Mountain Lake:

*We came together, and it seemed like we left as a big family. You know what I mean? It felt like there was a lot of—I shouldn’t say “broken,” but maybe forgotten relationships that were now open, or new ones were made.* (Staff 1)

### 3.2. Practicing Anishinaabe Knowledge

The Mountain Lake camp was planned intentionally to support the intergenerational learning and practice of Anishinaabe knowledge, including cultural skills, community history, and traditional responsibilities. The daily schedule involved a structured activity each morning, such as moccasin- and rattle-making, followed by a communal meal to teach food preparation. The participating youth and adults described how this hands-on practice provided an opportunity to learn directly from key knowledge holders with ongoing support from Elders. Although these Elders physically live in Biigtigong, opportunities for visiting cannot be assumed and concentrated time with them for land-based learning is especially rare:

*But I really enjoyed it. I guess that’s something I’ve always dreamed of doing is just spending time with Elders. I mean like I can go and visit with an Elder, but it’s not like we’ll make rattles together or make drums together, stuff like that.* (Youth 4)

The disconnect between youth and Elders was described as the result of age-related mobility challenges and a lack of confidence among some Elders in their abilities and experiences. Having a group of Elders at the camp was therefore critical for learning Anishinaabe knowledge, as the Elders encouraged each other, shared collectively, and filled in individual gaps in memory. As one staff member highlighted, the camp facilitated the practice of Anishinaabe knowledge among the older adults and Elders by bringing them on the land together:

*I don’t think [Elders] realize the value that they hold and that information, the stories, they don’t … And that’s what they don’t understand is just that importance. Just for them to be out there experiencing and listening to the language, and how that’s gonna help them unlock all that knowledge and language that they have within them.* (Staff 2)

Supported by each other, the Elders used the time at the camp to share their place-based knowledge of Mountain Lake with the younger participants, including personal, family, and community history in the area. One youth described how this learning was facilitated by being at the site with Elders to listen to their stories:

*Because they’ll start telling you stories about “Oh, we used to go swimming here. We used to swim here. Oh, this looks like this place.” They’ll tell you stories about how they lived in the bush and stuff. Yeah, I think it is. And they—Elders—I don’t know, it’s kinda weird, they always know trees or leaves and the berries and stuff. Like, “Oh, we used to pick these when we were little girls” and stuff.* (Youth 3)

Another young adult further explained that the Elders’ stories not only teach place-based knowledge, but Anishinaabe values, morals, and broader life lessons:

*You don’t know what you’re gonna learn from [Elders] today. It’s like, “Am I gonna learn some modesty or humility, or are they gonna tell me a funny story or something like that?” You never know what’s gonna come out of their mouths sometimes and it’s really nice.* (Staff 5)

The teachings from Elders and their shared life experiences became a source of social support for this individual to address ongoing emotional stress in their home life:

*You can have a bad day when you come home and then you can go out there and then like, you know, not fully vent, but you get some advice. And, you know, they get you level-headed and stuff like that. And then it kind of takes away from all that stress that was building up at home.* (Staff 5)

Finally, participants described how the camp activities facilitated Elders and youth to take up their traditional roles and responsibilities as Anishinaabe community members. As the Elders shared their knowledge and experiences, the youth practiced reciprocity by catering for the Elders’ needs, such as preparing their meals, making them tea, and helping them around the camp site. A staff member explained that while these roles used to be expected behaviour, many youth in the community have not grown up with teachings around how to interact with Elders:

*Overall, I was just like so impressed with the way our youth were able to help and assist. It took a lot of pushing, but considering that we haven’t really done activities like that with [Elders and youth] together. So, we can’t just assume that those youth know exactly what to do.* (Staff 2)

### 3.3. Fostering Community Pride

As camp participants strengthened their relationships with each other and the land at Mountain Lake, they described a renewed sense of belonging and pride as members of Biigtigong. For many participants, the camp activities reinforced a collective social and cultural identity grounded in the community stories, Anishinaabe teachings, and family histories shared by the Elders. Staff and youth explained how learning about personal and community ties to the Mountain Lake area had increased their understanding of who they are as Biigtigong Nishnaabeg, and their pride in being part of a long and multigenerational living history. One staff member summarized why listening to Elders’ stories around the campfire made them prouder to be a part of the community:

*It gives me a sense of identity in where I come from and who—Especially when you get one of those little Elders that you talk with and they know things about your family you don’t know. And it’s like wow, it just gives you who you are, a better understanding of where you’re grounding from, where you come from, who you are. And it just makes you feel more proud to be a part of the community when you hear all this stuff. I don’t know. I just love sitting there and listening to stories. I shouldn’t say stories. It’s history, right?* (Staff 1)

Similarly, all three groups of participants highlighted an activity to tie-dye Biigtigong shirts as meaningful for community pride. While not a cultural activity, the tie-dying was physically easy and accessible to all participants, allowing everyone to be involved. Using community shirts created a feeling of both group connectedness and, as one staff member expressed, that the group was representing the broader community in the reclamation of Mountain Lake:

*And doing it with community shirts too. I think that was the biggest hit for somebody, like for all those people walking away to be like, “I have a shirt that represents my community and I get to wear that.” So, I think that was really special to them too. I kind of seen a few of them all happy that they had got those shirts.* (Staff 3)

Processes of dispossession have largely disconnected Biigtigong from the Mountain Lake area and one of the key goals of the camp was to reassert community jurisdiction over this place. Camp participants explained that being part of this collective effort to reclaim Biigtigong’s territory instilled feelings of pride. For Elders, this pride was often related to witnessing how well previous generations had cared for Mountain Lake and then taking up their own responsibilities to this place:

*See the trees and all that? And that belongs to the Nishnaabeg, which is us guys. This is our land and look how good it’s been taken care of, for the last, probably, 40, 50 years. It’s so nice. It’s so pristine, you know?* (Elder 1)

For staff and youth, a renewed community pride came from learning about how large Biigtigong’s ancestral territory is, and how much land is available for community members to use and connect with. One staff member explained how decreasing land use and travel among families meant that many camp participants were surprised to know that Mountain Lake belongs to the community’s territory:

*I could see a lot of reclaiming going on and people taking pride in that because—when you seen people who came down to Mountain Lake—they were quite surprised like this was theirs, like this was something that you can enjoy. “This is for you.”* (Staff 4)

In particular, the act of reclaiming Mountain Lake reminded younger generations what the community is capable of when it asserts its power and fights for its rights. A youth participant expressed how community pride is related to knowing that Biigtigong is strong, resilient, and taking back its land, despite persistent colonial barriers and attitudes:

*For what our people have been through, we could have been moved or we could have been killed off or you know something like that. But we’re still walking on the same land that our ancestors did. To think that we’re still here and we’re resilient and we’re still fighting through all the stuff that the government throws at us. And not even the government, just people who don’t think, not highly of us, but they think lowly of us. And to think that—even just going to school—I’ve seen a lot of people and Indigenous people are such a low thought about person, thing. And just being able to connect on the land and the territory, it’s amazing to see what we’re fighting for.* (Youth 4)

## 4. Discussion

This paper qualitatively explored how processes of environmental repossession to reclaim and reconnect to land can foster community mental wellness. As part of a broader study to examine local meanings of repossession efforts, this research documented the experiences of community members participating in a land camp to repossess Mountain Lake in Biigtigong Nishnaabeg. Thematic analysis of story-based interviews with Biigtigong Elders, staff, and youth suggests that the Mountain Lake camp supported Anishinaabe identity and wellness through three important mechanisms: (1) strengthening intergenerational social relationships; (2) providing opportunities to share and practice Anishinaabe knowledge; and (3) fostering community belonging and pride.

The Mountain Lake camp was planned by the Department of Sustainable Development as a strategy of environmental repossession to assert Biigtigong’s Indigenous rights over this area. To reclaim Mountain Lake, the camp brought three generations of Biigtigong members together on the land for one week to reconnect to this place and each other. Through cultural and social activities, the camp facilitated intergenerational partnerships and sharing as a means to renew social relationships between Elders, adults, and youth. Although Biigtigong is a small community of approximately 500 on-reserve members, maintaining strong social relationships has been a challenge against historical and contemporary experiences of dispossession. Outside of daily routines and social constraints, such as technology, the camp provided participants with the time and space to overcome intergenerational tensions and reconnect. Throughout the week-long camp, the land of Mountain Lake supported this relational context as the source of shared connection between participants, their families, and ancestors. As one adult summarized, “We’re all connected through the people and the land. The people, the community bring us together, but it’s the land that keeps us together” (Staff 5). This strengthening of intergenerational community relationships increased both access to social support and facilitated a sense of belonging among participants.

Critical to the reclamation of Mountain Lake was the sharing of land-based Indigenous knowledge that imbues this site with meaning as an Anishinaabe place. The department staff planned the camp schedule to include both structured activities and flexible time to encourage the transmission of Anishinaabe knowledge between generations of participants. This intergenerational teaching was further encouraged by the growing community relationships and collective nature of learning at the camp, through which participants felt comfortable practicing their cultural roles as Anishinaabe community members. As Elders and youth took up and practiced their responsibilities as teachers and learners of Anishinaabe knowledge, they became increasingly confident in their skills and identity as Biigtigong Nishnaabeg, as well as their capacity to contribute to the community. In particular, Elders experienced an increased sense of self-worth related to the value and importance that other participants placed on their stories and teachings. 

Throughout the week, participants strengthened their connections with Mountain Lake, each other, and their Anishinaabe knowledge system. This led to the development of a shared sense of community pride. While all participants are band members of Biigtigong Nishnaabeg, some youth expressed not having strong social or cultural ties to this identity. In fact, as processes of dispossession have historically confined community members to their reserve, this colonial space has come to define many members’ senses of identity as Biigtigong Nishnaabeg, rather than their Anishinaabe ways of living. The process of repossession at Mountain Lake reminded staff and youth that their territory extends far beyond the small legal reserve and demonstrated that the community is exercising its power to reclaim the entire ancestral territory. Participants understood that the collective effort of environmental repossession relied on their individual participation. By showing up each day at the camp to listen to community stories, cook and share food, and canoe on the lake, they were each supporting the reclamation of Mountain Lake on behalf of the broader community. This shared awareness of purpose, strength, and connection fostered deep feelings of pride in the community, its culture, history, and identity. These feelings were reinforced through the production of a 2021 calendar with photographs and stories from the camp to share with all Biigtigong members.

Collective participation in the reclamation of Mountain Lake positively impacted community mental wellness. Framed by mino-bimaadiziwin and Anishinaabe relationships to land, the Mountain Lake camp was developed as a strategy of environmental repossession to reclaim Biigtigong’s territory and ways of living for a strong, healthy, and self-determining future. Through its various activities, including visiting and sharing stories, knowledge, and food, the Mountain Lake camp supported intergenerational connections to the land, each other, and to Anishinaabe knowledge. These intergenerational activities strengthened participants’ sense of social and cultural belonging with their Anishinaabe identity. All three generations experienced renewed relationships, pride, and community roles, which are important determinants of mental wellness for Indigenous youth, adults, and Elders [43,63,64,65]

Perhaps most importantly, the reclamation of Mountain Lake fostered a sense of collective empowerment and pride among participants. The act of coming together to assert Biigtigong’s rights to its territory was a demonstration of the community’s resilience and power, not only to the colonial state, but to the community members themselves. Against ongoing impacts of dispossession, the camp reinforced for participants the continuity of Biigtigong’s relationship to its territory, and the ways in which connection to land upholds their Anishinaabe identity and future. While research has drawn on the concepts of environmental dispossession and ecological grief to examine the complex mental health implications of land loss and disconnection [13,66,67], these results suggest that environmental repossession may be useful for understanding how land reconnection and self-determination can enhance Indigenous mental wellness, individually and collectively. Conceptually, these results advance an expanded understanding of how land camps and activities can support Indigenous mental wellness, as the act of land reclamation may represent an important aspect of healing for communities [23,24,51]. 

The outcomes of environmental repossession extend beyond land reoccupation and reclamation. As our results demonstrate, these processes restore the place-based relationships and knowledges that are foundational to Indigenous identities and conceptions of mental wellbeing. In this sense, applying environmental repossession is an inherently political act of resistance and resurgence in which communities assert self-determination not only over their lands, but also their collective wellness and futures. The land camp at Mountain Lake created the space for community members to gather and revitalize the roles, values, and relationships that connect each of them to their shared identity as Biigtigong Nishnaabeg. This practice of environmental repossession compliments Radu et al.’s [3] definition of healing as “political resistance and as a site of identity and cultural renegotiation” (p. 99). This is healing based not on biomedical understandings and practices, but those that defy the goals of colonization and encourage the return of Anishinaabe principles and ways of understanding wellness. In the wider context of Indigenous mental health research, there is a growing consensus that greater attention and resources must be allocated to land-based mental health programming and delivery [35,41,64]. These programs are culturally responsive to the unique traumas endured by Indigenous Peoples and communities—especially those related to processes of dispossession—and they offer dignified spaces of healing, with emphasis placed on relational practice, inclusion of traditional knowledge, and spirituality [34,48]. 

By exploring the links between land reclamation, identity, and community empowerment, our results suggest that healing and environmental repossession may be interconnected mechanisms of Indigenous decolonization. If decolonization is an active process of renewing Indigenous worldviews, knowledge systems, and ways of living, it requires the restoration of relationships to land and land-based responsibilities [57,68]. In Biigtigong, everyday practices of healing and environmental repossession are supporting this process as the community reclaims its territory and rebuilds its own practices of mental wellness along the pathway to mino-bimaadiziwin. At the camp, the everyday action of tie-dying a community shirt or frying bannock in the bush became a demonstration of Anishinaabe nationhood in that it fostered intergenerational belonging to community, culture, and the land [25,69,70].

## 5. Conclusions

In this paper, we examined the reclamation of Mountain Lake by Biigtigong Nishnaabeg in order to explore how processes of environmental repossession may foster mental wellness in Indigenous communities. Biigtigong’s Department of Sustainable Development planned a week-long camp at Mountain Lake as a means to reconnect community members to this land and the history and knowledge tied to this site. Our results demonstrate that for participating youth, staff, and Elders, the land camp strengthened social relationships, supported the sharing and practice of Anishinaabe knowledge, and fostered community pride in ways that reinforced Anishinaabe identity. In so doing, this study suggests an important link between Indigenous land reclamation, self-determination, and mental wellness and raises questions for future research around the implications of decolonization for physical, mental, emotional, and spiritual wellbeing. While research has broadly examined the relationship between community land tenure and wellbeing [71], as well as land connections and individual health [1], there is an opportunity to explore the specific strategies that communities are employing to assert their Indigenous rights to land and wellness.

Indigenous peoples globally are calling for ‘land back’ as they seek to address the ongoing structures of colonialism and revitalize self-determining nations. Every day Indigenous communities are living their relationships to land and developing new approaches, actions, and programs to strengthen their land-based identities. While many of these communities are based in their ancestral territories, such as Biigtigong and other reserve-based First Nations in Canada, equally important are the strategies of individuals, families, and groups who have been displaced from their homelands or may not be part of a formal community [40]. As Indigenous health geographers, we have a responsibility to use research processes to support Indigenous self-determination, not only by documenting the experiences of communities but by actively contributing to their goals through our time, skills, and financial resources.

## Figures and Tables

**Table 1:** Planned daily camp schedule.

	Monday	Tuesday	Wednesday	Thursday	Friday
Morning	Group introductions	Making rag moccasins with youth and elders	Making tie-dye shirts with youth and elders	Making rattles with youth and elders	Making medicine pouches with youth and elders
Lunch	Spaghetti and garlic bread	Indian tacos	Moose meat stew and dumplings	Fish fry	Macaroni soup and bannock
Afternoon	Community mapping Family tree mapping	Boating Canoeing Shoreline fishing Steamed dumplings	Storytelling Apple snacks (on the fire)	Boating Canoeing Shoreline fishing Rice pudding	Shoreline fishing Making pies

## Data Availability

The data presented in this study are not publicly available due to privacy concerns. The data are owned by the Department of Sustainable Development, Biigtigong Nishnaabeg.

## References

[1] Redvers J. (2020). “The land is a healer”: Perspectives on land-based healing from Indigenous practitioners in northern Canada. Int. J. Indig. Health.

[2] Ullrich J.S. (2019). For the love of our children: An Indigenous connectedness framework. Altern. Int. J. Indig. Peoples.

[3] Radu I., House L.M., Pashagumskum E. (2014). Land, life, and knowledge in Chisasibi: Intergenerational healing in the bush. Decolonization Indig. Educ. Soc..

[4] Robbins J.A., Dewar J. (2011). Traditional Indigenous approaches to healing and the modern welfare of traditional knowledge, spirituality and lands: A critical reflection on practices and policies taken from the Canadian Indigenous example. Int. Indig. Policy J..

[5] Bell N., Deer F., Falkenberg T. (2016). Mino-Bimaadiziwin: Education for the good life. Indigenous Perspectives on Education for Well-Being in Canada.

[6] McGuire P.D. (2013). Anishinaabe Giikeedaasiwin–Indigenous Knowledge: An Exploration of Resilience. Ph.D. Thesis.

[7] Debassige B. (2010). Re-conceptualizing Anishinaabe mino-bimaadiziwin (the good life) as research methodology: A spirit-centered way in Anishinaabe research. Can. J. Nativ. Educ..

[8] Styres S.D. (2017). Pathways for Remembering and Recognizing Indigenous Thought in Education: Philosophies of Lethi’nihstenha Ohwentsia’kekha (Land).

[9] Battiste M., Youngblood J. (2000). Protecting Indigenous Knowledge and Heritage: A Global Challenge.

[10] Richmond C.A.M., Big-Canoe K., Crooks V.A., Andrews G.J., Pearce J. (2018). Geographies of Indigenous Health. Routledge Handbook of Health Geography.

[11] Richmond C., Elliott S., Matthews R., Elliott B. (2005). The political ecology of health: Perceptions of environment, economy, health and well-being among ‘Namgis First Nation. Health Place.

[12] Wilson K. (2003). Therapeutic landscapes and First Nations peoples: An exploration of culture, health and place. Health Place.

[13] Lewis D., Castleden H., Apostle R., Francis S., Francis-Strickland K. (2021). Linking land displacement and environ-mental dispossession to Mi’kmaw health and well-being: Culturally relevant place-based interpretive frameworks matter. Can. Geogr./Géographe Can..

[14] Middleton J., Cunsolo A., Jones-Bitton A., Shiwak I., Wood M., Pollock N., Flowers C., Harper S.L. (2020). “We’re people of the snow”: Weather, climate change, and Inuit mental wellness. Soc. Sci. Med..

[15] Durkalec A., Furgal C., Skinner M.W., Sheldon T. (2015). Climate change influences on environment as a determinant of Indigenous health: Relationships to place, sea ice, and health in an Inuit community. Soc. Sci. Med..

[16] Richmond C.A., Ross N.A., Richmond C.A., Ross N.A. (2009). The determinants of First Nation and Inuit health: A critical population health approach. Health Place.

[17] Tobias J.K., Richmond C.A. (2014). “That land means everything to us as Anishinaabe….”: Environmental dispossession and resilience on the North Shore of Lake Superior. Health Place.

[18] Adelson N. (2005). The Embodiment of Inequity: Health Disparities in Aboriginal Canada. Can. J. Public Health.

[19] Simpson L.R. (2004). Anticolonial Strategies for the Recovery and Maintenance of Indigenous Knowledge. Am. Indian Q..

[20] Nightingale E., Richmond C.A. (2021). Reclaiming Mountain Lake: Applying environmental repossession in Biigtigong Nishnaabeg territory, Canada. Soc. Sci. Med..

[21] Hatala A.R., Morton D., Njeze C., Bird-Naytowhow K., Pearl T. (2019). Re-imagining miyo-wicehtowin: Human-nature relations, land-making, and wellness among Indigenous youth in a Canadian urban context. Soc. Sci. Med..

[22] Tobias J.K., Richmond C. (2016). Gimiigiwemin: Putting Knowledge Translation into Practice with Anishinaabe Communities. Int. J. Indig. Health.

[23] Big-Canoe K., Richmond C.A. (2014). Anishinabe youth perceptions about community health: Toward environmental repossession. Health Place.

[24] Pasternak S., King H., Yesno R. (2019). Land Back: A Yellowhead Institute Red Paper.

[25] Simpson L.B. (2017). As We Have Always Done: Indigenous Freedom through Radical Resistance.

[26] Mikraszewicz K., Richmond C. (2019). Paddling the Biigtig: Mino biimadisiwin practiced through canoeing. Soc. Sci. Med..

[27] Goodyear-Káōpua N. (2017). Protectors of the future, not protestors of the past: Indigenous Pacific activism and Mauna a Wākea. South Atl. Q..

[28] Hatala A.R., Njeze C., Morton D., Pearl T., Bird-Naytowhow K. (2020). Land and nature as sources of health and resilience among Indigenous youth in an urban Canadian context: A photovoice exploration. BMC Public Health.

[29] Hirsch R., Furgal C., Hackett C., Sheldon T., Bell T., Angnatok D., Winters K., Pamak C. (2016). Going Off, Growing Strong: A program to enhance individual youth and community resilience in the face of change in Nain, Nunatsiavut. Études/Inuit/Stud..

[30] Allen J., Hopper K., Wexler L., Kral M., Rasmus S., Nystad K. (2013). Mapping resilience pathways of Indigenous youth in five circumpolar communities. Transcult. Psychiatry.

[31] Nightingale E., Richmond C.A. (2022). Building structures of environmental repossession to reclaim land, self-determination and Indigenous wellness. Health Place.

[32] Duignan S., Moffat T., Martin-Hill D. (2022). Be like the running water: Assessing gendered and age-based water insecurity experiences with Six Nations First Nation. Soc. Sci. Med..

[33] Ahmed F., Zuk A., Tsuji L. (2021). The Impact of Land-Based Physical Activity Interventions on Self-Reported Health and Well-Being of Indigenous Adults: A Systematic Review. Int. J. Environ. Res. Public Health.

[34] Walsh R., Danto D., Sommerfeld J. (2020). Land-Based Intervention: A Qualitative Study of the Knowledge and Practices Associated with One Approach to Mental Health in a Cree Community. Int. J. Ment. Health Addict..

[35] Johnson-Jennings M., Billiot S., Walters K. (2020). Returning to Our Roots: Tribal Health and Wellness through Land-Based Healing. Genealogy.

[36] Restoule B.M., Hopkins C., Robinson J., Wiebe P.K. (2016). First Nations Mental Wellness: Mobilizing Change through Partnership and Collaboration. Can. J. Community Ment. Health.

[37] Waddell C.M., de Jager M.D., Gobeil J., Tacan F., Herron R.V., Allan J.A., Roger K. (2021). Healing journeys: Indig-enous Men’s reflections on resources and barriers to mental wellness. Soc. Sci. Med..

[38] Rowan M., Poole N., Shea B., Gone J.P., Mykota D., Farag M., Dell C. (2014). Cultural interventions to treat addic-tions in Indigenous populations: Findings from a scoping study. Subst. Abus. Treat. Prev. Policy.

[39] Stewart S.L. (2008). Promoting Indigenous mental health: Cultural perspectives on healing from Native counsellors in Canada. Int. J. Health Promot. Educ..

[40] Montesanti S., Fitzpatrick K., Fayant B., Pritchard C. (2022). Identifying priorities, directions and a vision for Indigenous mental health using a collaborative and consensus-based facilitation approach. BMC Health Serv. Res..

[41] Redvers N., Nadeau M., Prince D. (2021). Urban Land-Based Healing: A Northern Intervention Strategy. Int. J. Indig. Health.

[42] Fienup-Riordan A. (2001). “We talk to you because we love you”: Learning from Elders at culture camp. Anthropol. Humanism.

[43] Danto D., Walsh R., Sommerfeld J., Danto D., Zangeneh M. (2022). Learning from Those Who Do: Land-Based Healing in a Mushkegowuk Community. Indigenous Knowledge and Mental Health.

[44] Hickey J., Powling H., McKinney P., Robbins T., Carrier N., Nash A. (2020). “It’s a change your life kind of program”: A Healing-Focused Camping Weekend for Urban Indigenous Families Living in Fredericton, New Brunswick. First Peoples Child Fam. Rev..

[45] Mashford-Pringle A., Stewart S.L. (2019). Akiikaa (it is the land): Exploring land-based experiences with university students in Ontario. Glob. Health Promot..

[46] Healey G.K., Noah J., Mearns C. (2016). The Eight Ujarait (Rocks) Model: Supporting Inuit Adolescent Mental Health With an Intervention Model Based on Inuit Ways of Knowing. Int. J. Indig. Health.

[47] Lines L.-A., Jardine C.G., Yellowknives Dene First Nation Wellness Division (2019). Connection to the land as a youth-identified social determinant of Indigenous Peoples’ health. BMC Public Health.

[48] Gaudet J.C. (2021). Project George: An Indigenous Land-Based Approach to Resilience for Youth. Int. J. Indig. Health.

[49] Takano T. (2005). Connections with the land: Land-skills courses in Igloolik, Nunavut. Ethnography.

[50] Dobson C., Brazzoni R. (2016). Land based healing: Carrier First Nations’ addiction recovery program. J. Indige-Nous Wellbeing.

[51] Truth and Reconciliation Commission (TRC) of Canada (2015). The Final Report of the Truth and Reconciliation.

[52] De Leeuw S., Maurice S., Holyk T., Greenwood M., Adam W. (2012). With Reserves: Colonial Geographies and First Nations Health. Ann. Assoc. Am. Geogr..

[53] Tester F., Kulchyski P. (2011). Tammarniit (Mistakes): Inuit Relocation in the Eastern Arctic, 1939–1963.

[54] Bombay A., Matheson K., Anisman H. (2014). The intergenerational effects of Indian Residential Schools: Implications for the concept of historical trauma. Transcult. Psychiatry.

[55] Ohmagari K., Berkes F. (1997). Transmission of Indigenous Knowledge and Bush Skills Among the Western James Bay Cree Women of Subarctic Canada. Hum. Ecol..

[56] Ermine W., Nilson R., Sauchyn D., Sauve E., Smith R.Y. (2005). Isi Askiwan–the state of the land: Summary of the Prince Albert grand council Elders’ forum on climate change. J. Aborig. Health.

[57] Corntassel J. (2012). Re-envisioning resurgence. Decolonization Indig. Educ. Soc..

[58] Louis R.P. (2007). Can You Hear us Now? Voices from the Margin: Using Indigenous Methodologies in Geographic Research. Geogr. Res..

[59] Wilson S. (2008). Research is Ceremony: Indigenous Research Methods.

[60] Kovach M. (2009). Indigenous Methodologies: Characteristics, Conversations, and Contexts.

[61] McGregor D., McGregor D., Restoule J.P., Johnston R. (2018). Toward an Anishinaabe research paradigm: Theory and practice. Indigenous Research: Theories, Practices, and Relationships.

[62] Clarke V., Braun V., Hayfield N. (2015). Thematic analysis. Qualitative Psychology: A Practical Guide to Research Methods.

[63] Schill K., Terbasket E., Thurston W.E., Kurtz D., Page S., McLean F., Jim R., Oelke N. (2019). Everything is related and it all leads up to my mental well-being: A qualitative study of the determinants of mental wellness amongst urban indigenous elders. Br. J. Soc. Work..

[64] Snowshoe A., Crooks C.V., Tremblay P.F., Hinson R.E. (2017). Cultural Connectedness and Its Relation to Mental Wellness for First Nations Youth. J. Prim. Prev..

[65] Chandler M.J., Lalonde W.L., Greenwood M., de Leeuw S., Lindsay N.M., Reading C. (2015). Cultural wounds demand cultural medicines. Determinants of Indigenous Peoples’ Health in Canada: Beyond the Social.

[66] Cunsolo W.A., Stephenson E., Allen J., Bourque F., Drossos A., Elgarøy S., Kral M., Mauro I., Moses J., Pearce T. (2015). Examining relationships between climate change and mental health in the Circumpolar North [Original Paper]. Reg. Environ. Change.

[67] Simpson L., DaSilva J., Riffel B., Sellers P. (2009). The Responsibilities of Women: Confronting Environmental Contami-nation in the Traditional Territories of Asubpeechoseewagong Netum Anishinabek (Grassy Narrows) and Wabauskang First Nation. Int. J. Indig. Health.

[68] Tuck E., Yang K.W. (2012). Decolonization is not a metaphor. Decolonization Indig. Educ. Soc..

[69] Corntassel J., Hardbarger T. (2019). Educate to perpetuate: Land-based pedagogies and community resurgence. Int. Rev. Educ..

[70] Adelson N. (2000). ‘Being Alive Well’ Health and Politics of Cree Well-Being.

[71] Fligg R.A., Robinson D.T. (2020). Reviewing First Nation land management regimes in Canada and exploring their relationship to community well-being. Land Use Policy.

